# (*E*)-3-(4-Chloro­phen­yl)-1-(2-fur­yl)prop-2-en-1-one

**DOI:** 10.1107/S1600536808021934

**Published:** 2008-07-19

**Authors:** Hoong-Kun Fun, P. S. Patil, Samuel Robinson Jebas, S. M. Dharmaprakash

**Affiliations:** a X-ray Crystallography Unit, School of Physics, Universiti Sains Malaysia, 11800 USM, Penang, Malaysia; b Department of Studies in Physics, Mangalore University, Mangalagangotri, Mangalore 574 199, India

## Abstract

In the title mol­ecule, C_13_H_9_ClO_2_, the benzene and furyl rings are slightly twisted from each other with a dihedral angle of 5.1 (1)°. An intra­molecular C—H⋯O hydrogen-bond inter­action generates an *S*(5) ring motif. In the crystal structure, mol­ecules are stacked along the *b* axis and the crystal packing is stabilized by weak inter­molecular C—H⋯O hydrogen bonds.

## Related literature

For related literature on the biological and nonlinear optical properties of chalcone derivatives, see: Agrinskaya *et al.* (1999 ▶); Chopra *et al.* (2007 ▶); DiCesare & Lakowicz (2000 ▶); Patil *et al.* (2006 ▶, 2007 ▶); Gu, Ji, Patil & Dharmaprakash (2008 ▶); Gu, Ji, Patil, Dharmaprakash & Wang (2008 ▶). For bond-length data, see: Allen *et al.* (1987 ▶). For graph-set analysis of hydrogen bonding, see: Bernstein *et al.* (1995 ▶).
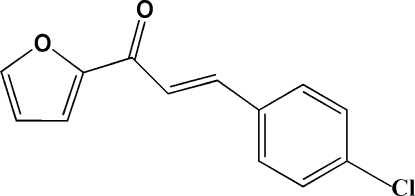


## Experimental

### 

#### Crystal data

C_13_H_9_ClO_2_
*M* *_r_* = 232.65
Orthorhombic, 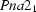

*a* = 21.3399 (7) Å
*b* = 3.7912 (1) Å
*c* = 12.9444 (4) Å
*V* = 1047.25 (5) Å^3^
*Z* = 4
Mo *K*α radiation
μ = 0.34 mm^−1^
*T* = 100.0 (1) K
0.40 × 0.29 × 0.21 mm

#### Data collection

Bruker SMART APEXII CCD area-detector diffractometer
Absorption correction: multi-scan (*SADABS*; Bruker, 2005 ▶) *T*_min_ = 0.875, *T*_max_ = 0.931
13568 measured reflections
5209 independent reflections
4211 reflections with *I* > 2σ(*I*)
*R*_int_ = 0.025

#### Refinement

*R*[*F* ^2^ > 2σ(*F* ^2^)] = 0.047
*wR*(*F* ^2^) = 0.126
*S* = 1.08
5209 reflections
145 parameters
1 restraint
H-atom parameters constrained
Δρ_max_ = 0.58 e Å^−3^
Δρ_min_ = −0.28 e Å^−3^
Absolute structure: Flack (1983 ▶), 2227 Friedel pairs
Flack parameter: 0.07 (6)

### 

Data collection: *APEX2* (Bruker, 2005 ▶); cell refinement: *APEX2*; data reduction: *SAINT* (Bruker, 2005 ▶); program(s) used to solve structure: *SHELXTL* (Sheldrick, 2008 ▶); program(s) used to refine structure: *SHELXTL*; molecular graphics: *SHELXTL*; software used to prepare material for publication: *SHELXTL* and *PLATON* (Spek, 2003 ▶).

## Supplementary Material

Crystal structure: contains datablocks global, I. DOI: 10.1107/S1600536808021934/lh2657sup1.cif

Structure factors: contains datablocks I. DOI: 10.1107/S1600536808021934/lh2657Isup2.hkl

Additional supplementary materials:  crystallographic information; 3D view; checkCIF report

## Figures and Tables

**Table 1:** Hydrogen-bond geometry (Å, °)

*D*—H⋯*A*	*D*—H	H⋯*A*	*D*⋯*A*	*D*—H⋯*A*
C7—H7*A*⋯O2	0.93	2.52	2.8411 (17)	101
C13—H13*A*⋯O2^i^	0.93	2.48	3.2535 (18)	140

Symmetry code: (i) 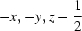.

**Fig. 1.**
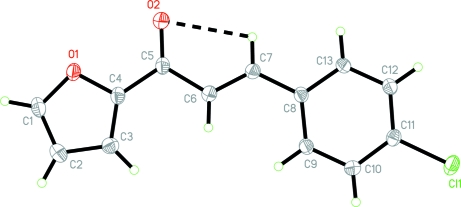
The molecular structure of the title compound, showing 50% probability displacement ellipsoids and the atom numbering scheme. The dashed line indicates a hydrogen bond.

**Fig. 2.**
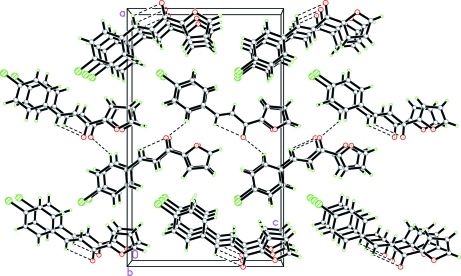
The crystal packing of the title compound, viewed along the b axis. Hydrogen bonds are shown as dashed lines.

## supplementary crystallographic information

### Comment

Chalcone derivatives continue to attract the interest of chemists, biologists and physicists due to their remarkable biological and nonlinear optical properties (Chopra *et al.*, 2007; DiCesare & Lakowicz, 2000; Patil, *et al.*, 2006, 2007; Agrinskaya *et al.*, 1999; Gu, Ji, Patil & Dharmaprakash, 2008; Gu, Ji, Patil, Dharmaprakash & Wang, 2008). We have synthesized the title compound (I) and its structure is reported here.

The bond lengths and bond angles in (I) have normal values (Allen *et al.*, 1987). The benzene and furyl rings in the molecule are essentially planar with the maximum deviation from planarity being -0.003 (18)Å for atom C12 and -0.004 (14)Å for atom O1 respectively. The dihedral angle between the benzene and the furyl rings is 5.1 (1)°, indicating that they are only slightly twisted from each other.

An intramolecular C—H···O hydrogen bond generates an S(5) ring motif (Bernstein *et al.*, 1995). In the crystal structure, the molecules are are stacked along the *b* axis. The crystal packing is consolidated by C—H···O hydrogen bond interactions.

### Experimental

The compound (I) was synthesized by the condensation of 4 -chlorobenzaldehyde (0.01 mol, 1.49 g m) with 2-acetylfuran (0.01 mol, 1.01 ml) in methanol (60 ml) in the presence of a catalytic amount of sodium hydroxide solution (5 ml, 30%). After stirring (6 h), the contents of the flask were poured into ice-cold water (500 ml) and left to stand for 5 h. The resulting crude solid was filtered and dried. Then precipitated compound was recrystallized from N, *N*-dimethylformamide (DMF).

### Refinement

H atoms were positioned geometrically [C—H = 0.93 Å] and refined using a riding model, with *U*_iso_(H) = 1.2*U*_eq_(C).

### Crystal data

**Table:**

C_13_H_9_ClO_2_	*F*_000_ = 480
*M**_r_* = 232.65	*D*_x_ = 1.476 Mg m^−^^3^
Orthorhombic, *P**n**a*2_1_	Mo *K*α radiation λ = 0.71073 Å
Hall symbol: P 2c -2n	Cell parameters from 4886 reflections
*a* = 21.3399 (7) Å	θ = 2.5–37.2º
*b* = 3.79120 (10) Å	µ = 0.34 mm^−^^1^
*c* = 12.9444 (4) Å	*T* = 100.0 (1) K
*V* = 1047.25 (5) Å^3^	Block, colourless
*Z* = 4	0.40 × 0.29 × 0.21 mm

### Data collection

**Table:**

Bruker SMART APEXII CCD area-detector diffractometer	5209 independent reflections
Radiation source: fine-focus sealed tube	4211 reflections with *I* > 2σ(*I*)
Monochromator: graphite	*R*_int_ = 0.025
*T* = 100.0(1) K	θ_max_ = 38.2º
φ and ω scans	θ_min_ = 2.5º
Absorption correction: multi-scan(SADABS; Bruker, 2005)	*h* = −31→37
*T*_min_ = 0.875, *T*_max_ = 0.931	*k* = −6→6
13568 measured reflections	*l* = −22→19

### Refinement

**Table:**

Refinement on *F*^2^	Hydrogen site location: inferred from neighbouring sites
Least-squares matrix: full	H-atom parameters constrained
*R*[*F*^2^ > 2σ(*F*^2^)] = 0.047	*w* = 1/[σ^2^(*F*_o_^2^) + (0.0686*P*)^2^] where *P* = (*F*_o_^2^ + 2*F*_c_^2^)/3
*wR*(*F*^2^) = 0.126	(Δ/σ)_max_ < 0.001
*S* = 1.08	Δρ_max_ = 0.58 e Å^−^^3^
5209 reflections	Δρ_min_ = −0.27 e Å^−^^3^
145 parameters	Extinction correction: none
1 restraint	Absolute structure: Flack (1983), 2216 Friedel pairs
Primary atom site location: structure-invariant direct methods	Flack parameter: 0.07 (6)
Secondary atom site location: difference Fourier map	

### Special details

**Table:**

Experimental. The data was collected with the Oxford Cyrosystem Cobra low-temperature attachment.
Geometry. All e.s.d.'s (except the e.s.d. in the dihedral angle between two l.s. planes) are estimated using the full covariance matrix. The cell e.s.d.'s are taken into account individually in the estimation of e.s.d.'s in distances, angles and torsion angles; correlations between e.s.d.'s in cell parameters are only used when they are defined by crystal symmetry. An approximate (isotropic) treatment of cell e.s.d.'s is used for estimating e.s.d.'s involving l.s. planes.
Refinement. Refinement of *F*^2^ against ALL reflections. The weighted *R*-factor *wR* and goodness of fit *S* are based on *F*^2^, conventional *R*-factors *R* are based on *F*, with *F* set to zero for negative *F*^2^. The threshold expression of *F*^2^ > σ(*F*^2^) is used only for calculating *R*-factors(gt) *etc*. and is not relevant to the choice of reflections for refinement. *R*-factors based on *F*^2^ are statistically about twice as large as those based on *F*, and *R*- factors based on ALL data will be even larger.

### Fractional atomic coordinates and isotropic or equivalent isotropic displacement parameters (Å^2^)

**Table:**

	*x*	*y*	*z*	*U*_iso_*/*U*_eq_	
Cl1	0.249271 (17)	0.53749 (9)	0.21078 (5)	0.02637 (9)	
O1	0.03179 (5)	0.2805 (3)	0.94149 (7)	0.0225 (2)	
O2	0.00152 (6)	0.1062 (3)	0.74067 (9)	0.0266 (2)	
C1	0.06091 (7)	0.3896 (4)	1.02879 (11)	0.0246 (3)	
H1A	0.0454	0.3557	1.0952	0.030*	
C2	0.11562 (8)	0.5548 (4)	1.00653 (12)	0.0243 (3)	
H2A	0.1437	0.6536	1.0533	0.029*	
C3	0.12135 (7)	0.5463 (4)	0.89712 (12)	0.0215 (3)	
H3A	0.1540	0.6388	0.8581	0.026*	
C4	0.06937 (7)	0.3752 (4)	0.86037 (10)	0.0192 (2)	
C5	0.04991 (6)	0.2723 (4)	0.75621 (9)	0.0198 (2)	
C6	0.09349 (7)	0.3737 (4)	0.67253 (10)	0.0202 (2)	
H6A	0.1274	0.5190	0.6875	0.024*	
C7	0.08493 (6)	0.2600 (4)	0.57564 (10)	0.0188 (2)	
H7A	0.0496	0.1223	0.5633	0.023*	
C8	0.12568 (6)	0.3314 (4)	0.48732 (9)	0.0178 (2)	
C9	0.18485 (6)	0.4918 (4)	0.49906 (11)	0.0188 (2)	
H9A	0.1984	0.5579	0.5645	0.023*	
C10	0.22299 (7)	0.5524 (4)	0.41438 (11)	0.0192 (2)	
H10A	0.2621	0.6577	0.4223	0.023*	
C11	0.20160 (7)	0.4524 (4)	0.31741 (11)	0.0186 (2)	
C12	0.14356 (7)	0.2960 (4)	0.30286 (10)	0.0198 (2)	
H12A	0.1301	0.2333	0.2370	0.024*	
C13	0.10594 (6)	0.2350 (4)	0.38834 (9)	0.0185 (2)	
H13A	0.0670	0.1284	0.3797	0.022*	

### Atomic displacement parameters (Å^2^)

**Table:**

	*U*^11^	*U*^22^	*U*^33^	*U*^12^	*U*^13^	*U*^23^
Cl1	0.02847 (16)	0.02869 (17)	0.02196 (14)	−0.00201 (13)	0.00812 (11)	0.00173 (17)
O1	0.0200 (4)	0.0321 (6)	0.0156 (4)	0.0000 (4)	0.0017 (3)	0.0021 (4)
O2	0.0241 (5)	0.0361 (6)	0.0196 (4)	−0.0065 (4)	0.0001 (4)	0.0017 (4)
C1	0.0265 (7)	0.0314 (8)	0.0161 (5)	0.0055 (6)	−0.0005 (5)	−0.0023 (5)
C2	0.0268 (7)	0.0249 (7)	0.0211 (6)	0.0033 (5)	−0.0045 (5)	−0.0027 (5)
C3	0.0201 (6)	0.0220 (7)	0.0223 (6)	0.0000 (5)	−0.0018 (5)	0.0026 (5)
C4	0.0201 (5)	0.0218 (6)	0.0157 (5)	0.0029 (5)	0.0008 (4)	0.0019 (4)
C5	0.0204 (6)	0.0236 (6)	0.0153 (5)	0.0028 (5)	0.0010 (4)	0.0013 (4)
C6	0.0196 (5)	0.0224 (6)	0.0185 (5)	−0.0008 (5)	0.0010 (4)	0.0006 (5)
C7	0.0186 (5)	0.0197 (6)	0.0181 (5)	−0.0006 (5)	0.0004 (4)	0.0011 (4)
C8	0.0171 (5)	0.0209 (6)	0.0153 (5)	0.0020 (5)	−0.0006 (4)	−0.0002 (4)
C9	0.0191 (5)	0.0212 (6)	0.0160 (5)	0.0004 (5)	−0.0007 (4)	−0.0011 (4)
C10	0.0181 (6)	0.0187 (6)	0.0208 (5)	−0.0012 (5)	−0.0005 (4)	0.0003 (5)
C11	0.0209 (6)	0.0166 (6)	0.0185 (5)	0.0013 (5)	0.0031 (4)	0.0016 (4)
C12	0.0217 (6)	0.0212 (6)	0.0164 (5)	−0.0006 (5)	−0.0006 (4)	−0.0014 (4)
C13	0.0173 (5)	0.0218 (6)	0.0164 (5)	−0.0001 (5)	−0.0023 (4)	0.0004 (4)

### Geometric parameters (Å, °)

**Table:**

Cl1—C11	1.7446 (14)	C6—H6A	0.9300
O1—C1	1.3543 (18)	C7—C8	1.4617 (18)
O1—C4	1.3691 (16)	C7—H7A	0.9300
O2—C5	1.2261 (18)	C8—C13	1.3974 (17)
C1—C2	1.356 (2)	C8—C9	1.410 (2)
C1—H1A	0.9300	C9—C10	1.384 (2)
C2—C3	1.422 (2)	C9—H9A	0.9300
C2—H2A	0.9300	C10—C11	1.388 (2)
C3—C4	1.370 (2)	C10—H10A	0.9300
C3—H3A	0.9300	C11—C12	1.386 (2)
C4—C5	1.4637 (18)	C12—C13	1.3865 (18)
C5—C6	1.4786 (18)	C12—H12A	0.9300
C6—C7	1.3388 (18)	C13—H13A	0.9300
			
C1—O1—C4	106.93 (11)	C6—C7—H7A	116.9
O1—C1—C2	111.03 (13)	C8—C7—H7A	116.9
O1—C1—H1A	124.5	C13—C8—C9	118.80 (12)
C2—C1—H1A	124.5	C13—C8—C7	119.30 (12)
C1—C2—C3	105.97 (14)	C9—C8—C7	121.90 (11)
C1—C2—H2A	127.0	C10—C9—C8	120.85 (12)
C3—C2—H2A	127.0	C10—C9—H9A	119.6
C4—C3—C2	106.68 (14)	C8—C9—H9A	119.6
C4—C3—H3A	126.7	C9—C10—C11	118.51 (12)
C2—C3—H3A	126.7	C9—C10—H10A	120.7
O1—C4—C3	109.39 (12)	C11—C10—H10A	120.7
O1—C4—C5	118.06 (12)	C12—C11—C10	122.24 (12)
C3—C4—C5	132.51 (13)	C12—C11—Cl1	119.52 (11)
O2—C5—C4	121.81 (12)	C10—C11—Cl1	118.23 (11)
O2—C5—C6	122.90 (13)	C11—C12—C13	118.71 (12)
C4—C5—C6	115.27 (12)	C11—C12—H12A	120.6
C7—C6—C5	121.11 (13)	C13—C12—H12A	120.6
C7—C6—H6A	119.4	C12—C13—C8	120.89 (12)
C5—C6—H6A	119.4	C12—C13—H13A	119.6
C6—C7—C8	126.29 (13)	C8—C13—H13A	119.6
			
C4—O1—C1—C2	0.69 (17)	C5—C6—C7—C8	−177.75 (13)
O1—C1—C2—C3	−0.41 (18)	C6—C7—C8—C13	−171.24 (14)
C1—C2—C3—C4	−0.02 (18)	C6—C7—C8—C9	9.4 (2)
C1—O1—C4—C3	−0.69 (16)	C13—C8—C9—C10	−0.2 (2)
C1—O1—C4—C5	177.19 (12)	C7—C8—C9—C10	179.11 (14)
C2—C3—C4—O1	0.44 (17)	C8—C9—C10—C11	0.2 (2)
C2—C3—C4—C5	−177.02 (15)	C9—C10—C11—C12	0.2 (2)
O1—C4—C5—O2	0.0 (2)	C9—C10—C11—Cl1	178.75 (11)
C3—C4—C5—O2	177.28 (16)	C10—C11—C12—C13	−0.6 (2)
O1—C4—C5—C6	−178.30 (12)	Cl1—C11—C12—C13	−179.09 (11)
C3—C4—C5—C6	−1.0 (2)	C11—C12—C13—C8	0.5 (2)
O2—C5—C6—C7	−6.2 (2)	C9—C8—C13—C12	−0.2 (2)
C4—C5—C6—C7	172.11 (14)	C7—C8—C13—C12	−179.49 (13)

### Hydrogen-bond geometry (Å, °)

**Table:**

*D*—H···*A*	*D*—H	H···*A*	*D*···*A*	*D*—H···*A*
C7—H7A···O2	0.93	2.52	2.8411 (17)	101
C13—H13A···O2^i^	0.93	2.48	3.2535 (18)	140

Symmetry codes: (i) −*x*, −*y*, *z*−1/2.
